# Spatiotemporal Dynamics of Dexmedetomidine-Induced Electroencephalogram Oscillations

**DOI:** 10.1371/journal.pone.0163431

**Published:** 2016-10-06

**Authors:** Oluwaseun Akeju, Seong-Eun Kim, Rafael Vazquez, James Rhee, Kara J. Pavone, Lauren E. Hobbs, Patrick L. Purdon, Emery N. Brown

**Affiliations:** 1 Department of Anesthesia, Critical Care and Pain Medicine, Massachusetts General Hospital, Harvard Medical School, Boston, MA, United States of America; 2 Department of Brain and Cognitive Science, Massachusetts Institute of Technology, Cambridge, MA, United States of America; University of California Los Angeles, UNITED STATES

## Abstract

An improved understanding of the neural correlates of altered arousal states is fundamental for precise brain state targeting in clinical settings. More specifically, electroencephalogram recordings are now increasingly being used to relate drug-specific oscillatory dynamics to clinically desired altered arousal states. Dexmedetomidine is an anesthetic adjunct typically administered in operating rooms and intensive care units to produce and maintain a sedative brain state. However, a high-density electroencephalogram characterization of the neural correlates of the dexmedetomidine-induced altered arousal state has not been previously accomplished. Therefore, we administered dexmedetomidine (1mcg/kg bolus over 10 minutes, followed by 0.7mcg/kg/hr over 50 minutes) and recorded high-density electroencephalogram signals in healthy volunteers, 18–36 years old (n = 8). We analyzed the data with multitaper spectral and global coherence methods. We found that dexmedetomidine was associated with increased slow-delta oscillations across the entire scalp, increased theta oscillations in occipital regions, increased spindle oscillations in frontal regions, and decreased beta oscillations across the entire scalp. The theta and spindle oscillations were globally coherent. During recovery from this state, these electroencephalogram signatures reverted towards baseline signatures. We report that dexmedetomidine-induced electroencephalogram signatures more closely approximate the human sleep onset process than previously appreciated. We suggest that these signatures may be targeted by real time visualization of the electroencephalogram or spectrogram in clinical settings. Additionally, these signatures may aid the development of control systems for principled neurophysiological based brain-state targeting.

## Introduction

Electroencephalogram recordings are now increasingly being used to relate drug-specific oscillatory dynamics to clinically desired altered arousal states [1]. In this investigation, we performed a systematic neural oscillation based characterization of dexmedetomidine, an alpha2-adrenoceptor agonist typically used to induce a sedative brain state in operating rooms and intensive care units (ICUs). Preclinical studies suggest that dexmedetomidine exerts its effects by blocking the release of norepinephrine from the locus ceruleus (LC) in the pons, resulting in loss of adrenergic inputs to the ventral lateral pre-optic (VLPO) nucleus of the hypothalamus [2–7]. Downstream activation of the VLPO and its subsequent inhibition of midbrain and pontine arousal centers are postulated to be one of the ways in which both non-REM sleep and the dexmedetomidine-induced brain state are initiated [8]. Thus, in contrast to other anesthetic agents, dexmedetomidine may very closely approximate and confer some benefits of non-REM sleep.

Clinical studies have described spindle (12–16 Hz) oscillations in frontal electroencephalogram electrode channels during dexmedetomidine-induced sedation. This suggests that the dexmedetomidine-induced altered arousal state shares similarities with non-REM II sleep [9, 10]. However, spindle oscillations have also been described during surgical levels of unconsciousness [11, 12] and during anesthesia-induced burst-suppression [13]. This arises likely due to ambiguity in clearly defining spindle oscillations. Thus, it is unclear whether basic science studies performed in laboratory animals that suggest dexmedetomidine activates the VLPO—and may approximate or confer some benefits of sleep—is translatable to humans. However, a systematic characterization of all dexmedetomidine-induced neural oscillations may assist us in drawing parallels between sleep states and this drug-induced brain state. These parallels are essential to ongoing efforts geared at refining sedation regimens—to reduce the morbidity associated with sedatives—in critically ill patients such that physiological states (i.e. non-REM sleep) may be approximated when appropriate.

Marzano et al. recently characterized the neural oscillations associated with human sleep onset, which was defined as the first epoch of non REM II sleep and found increased slow-delta (0.5–4 Hz) oscillations across the entire scalp, occipital theta (5–7 Hz) oscillations, spindle (12–15 Hz) oscillations in centro-parietal regions, and decreased beta (18–25 Hz) oscillation across the entire scalp [14]. Since basic science evidence suggests that dexmedetomidine engages endogenous sleep mechanisms, we therefore hypothesized that the spatiotemporal dynamics of neural oscillations induced by dexmedetomidine should parallel those described for human sleep onset. To explore this hypothesis, we measured and analyzed dexmedetomidine-induced electroencephalogram signals obtained from healthy volunteers (n = 8), 18 to 36 years of age.

## Materials and Methods

### Patient Selection and Data Collection

As described in detail previously [10, 15], we measured 64-channel electroencephalogram during baseline, dexmedetomidine-induced altered arousal and recovery in 8 healthy volunteers (5 males), with an average age of 26.6 (SD: 3.8), and an average weight of 65.3 (8.6) kg. We defined baseline as an awake, eyes closed period prior to the administration of dexmedetomidine. During this period response to the auditory stimuli was used to confirm that study subjects were awake. The Human Research Committee at the Massachusetts General Hospital approved this study. All subjects provided written informed consent and were American Society of Anesthesiology Physical Status I with Mallampati Class I airway anatomy. In addition to standard pre-anesthesia assessments, a urine toxicology screen was performed to ensure that subjects had not taken drugs that might confound the electroencephalogram or behavioral results. We administered a urine pregnancy test for each female subject to confirm that they were not pregnant. Before the start of the study, we required subjects to take nothing by mouth for at least 8 hours. Dexmedetomidine was administered as a 1mcg/kg loading bolus over 10 minutes, followed by a 0.7mcg/kg/hr infusion for 50 minutes. We monitored each subject’s heart rate with 5 lead electrocardiogram, oxygen saturation through pulse oximetry, respiration and expired carbon dioxide with capnography, and blood pressure with a standard non-invasive cuff. We recorded the electroencephalogram using a 64-channel BrainVision MRI Plus system (Brain Products, Gilching, Germany) with a sampling rate of 1,000 Hz, resolution 0.5 V least significant bit, bandwidth 0.001–250 Hz. Volunteers were instructed to close their eyes throughout the study to avoid eye-blink artifacts in the electroencephalogram. Volunteers were presented with auditory stimuli during the study and asked to respond by button presses to assess the level of conscious behavior. The stimuli consisted of the volunteer’s name presented every two minutes. A period of two minutes was chosen to ensure that repeated auditory tasks were not sufficiently arousing. A limited analysis of two frontal electrodes (per subject) from this dataset has previously been published [10].

### Electroencephalogram Preprocessing and Epoch Selection

We applied an anti-aliasing filter and down-sampled the electroencephalogram data to 250 Hz before analysis. Electroencephalogram signals were re-montaged to a nearest-neighbor Laplacian reference, using distances along the scalp surface to weigh neighboring electrode contributions [16]. First, 2-minute electroencephalogram segments were selected from all subjects during the awake, eyes closed baseline. Eye closure facilitates distinguishing between normal awake, eyes-closed occipital alpha oscillations and the frontal alpha oscillations associated with anesthesia induced altered arousal [16]. Electroencephalogram data segments during the dexmedetomidine induced brain state were selected based on the behavioral response. For the dexmedetomidine-induced brain state, the first artifact free electroencephalogram epoch after a series of at least three successive failures (6-minutes) to respond to the auditory task was chosen. The chosen epochs occurred after the bolus dose of dexmedetomidine. For the recovery brain state, the first artifact free electroencephalogram epoch after a series of at least three successive responses (6-minutes) was chosen. The chosen EEG epochs were obtained after the induction bolus of dexmedetomidine, during a stable maintenance infusion of dexmedetomidine.

### Spectral Analysis

Spectral analysis was performed using multitaper spectral estimation methods [17]. We computed spectra and spectrograms with window lengths of T = 2 seconds with 1.9 second overlap, time-bandwidth product TW = 2, number of tapers K = 3 and spectral resolution of 2 Hz in the Chronux toolbox (http://chronux.org). The spectrum of frequencies over time (i.e., spectrograms) within the 0.001 to 30Hz range was plotted for individual electrodes in each subject. Group-level spectrograms were computed by taking the median across subjects. For spatial distribution of spectral power over the scalp, we placed group-median spectrograms at each recording electrode location (44 electrodes).

### Topographic Analysis

Scalp power distributions of specific frequency bands were computed using interpolation of the electrode montage with the topoplot function in EEGLab [18]. We computed group-averaged spectra by taking the mean of group-level spectrograms across the entire epochs (2 minutes) and then averaged them over each frequency band of interest for each electrode.

### Global Coherence Analysis

Global coherence quantifies predominance of the largest eigenvalue in the eigenvalue decomposition of the cross spectral matrix. The parameters for the global coherence analysis are TW = 3, K = 5, T = 4 seconds and a non-overlapping window, yielding a spectral resolution of 1.5 Hz. For each non-overlapping window, we computed the cross-spectral matrix using multitaper methods for each subject, and took the median over whole windows of the real and imaginary parts of the cross-spectral matrix for each subject. We applied then the eigenvalue decomposition to the cross-spectral matrix at each frequency. The cross-spectral matrix at each frequency S(f) can be factorized as

$$\text{S}\left(\text{f}\right)=\text{U}\left(\text{f}\right)\Lambda \left(\text{f}\right)\text{U}{\left(\text{f}\right)}^{\text{H}}$$

where U^H^ is the complex conjugate transpose of U and a unitary matrix whose ith column is the eigenvector u_i_, and is the diagonal matrix whose diagonal elements are the corresponding eigenvalues, _ii_ = _i_. The global coherence is the ratio of the largest eigenvalue to the sum of eigenvalues [19]:

$${\text{C}}_{\text{global}}\left(\text{f}\right)=\left(\lambda_{\text{max}}\left(\text{f}\right)\right)/\left(\sum \;\lambda_{\text{i}}\left(\text{f}\right)\right).$$

When the largest eigenvalue is large compared with the remaining ones, the principal mode is dominant where the eigenvector u_max_ corresponding to the largest eigenvalue _max_ at a given frequency is defined as the principal mode of oscillation for that frequency. The group-level global coherence was calculated by taking a median across subjects. The absolute value of principal mode describes a coherent spatial distribution over all electrode sites. Group-level scalp coherence distributions were computed by taking the median across subjects and the topographical distribution were computed using the topoplot function in EEGLab.

### Statistical Analysis

To assess statistical significance for the difference in spectra at each frequency, we derived frontal and occipital electrodes to better reflect signals unique to frontal and occipital scalp locations. To derive these electrodes, we averaged 5 frontal electrode (FPZ, FP1, FP2,AF3, AF4) and 5 occipital electrodes (Oz, O1, O2, PO3, PO4) and computed the 95% confidence interval by using a median bootstrap algorithm. We randomly selected spectra with replacement from spectrograms over all time windows at each frequency to regenerate the spectrogram. We took the median from the regenerated spectrogram over time for each subject. We calculated differences between two median spectra across different states or regions for each subject, and took a median difference across subjects. We repeated this procedure 2000 times and calculated the 95% confidence interval of the median difference at each frequency.

## Results

### Scalp Spatiotemporal Representation of Dexmedetomidine-Induced Oscillations

**Fig 1:**
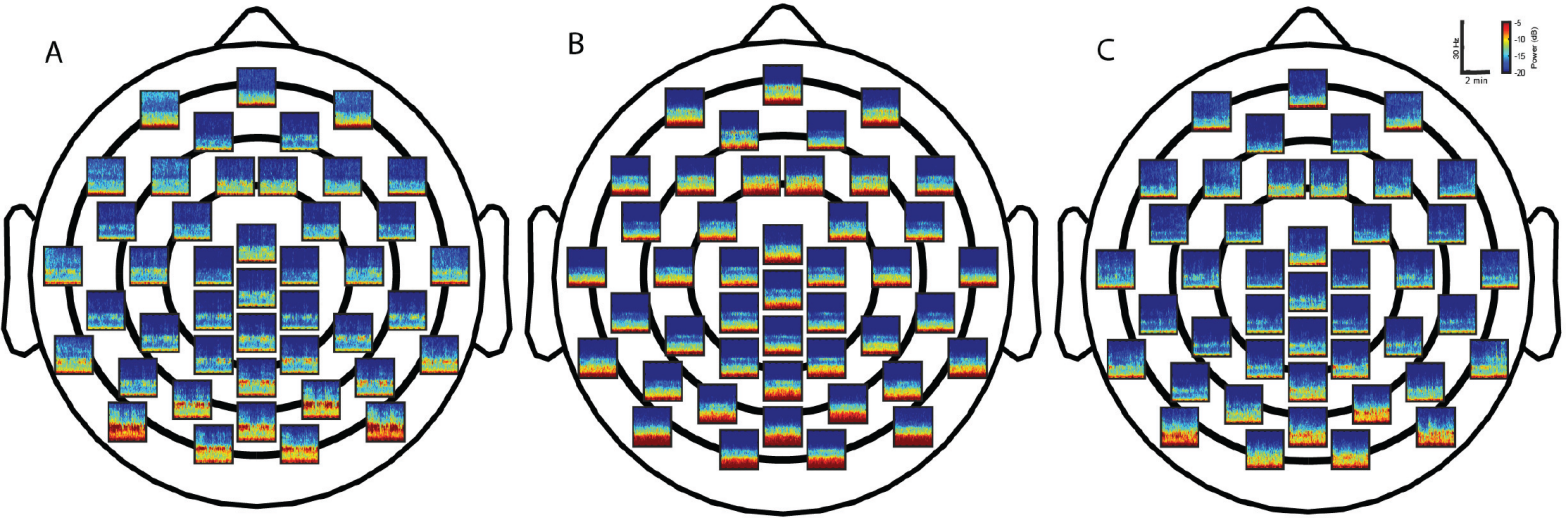
Spatial distribution of spectral power. Group-median spectrograms at each recording electrode location across the scalp in study volunteers (n = 8). (A) Baseline. (B) Dexmedetomidine-induced altered arousal. (C) Recovery.

**Fig 2:**
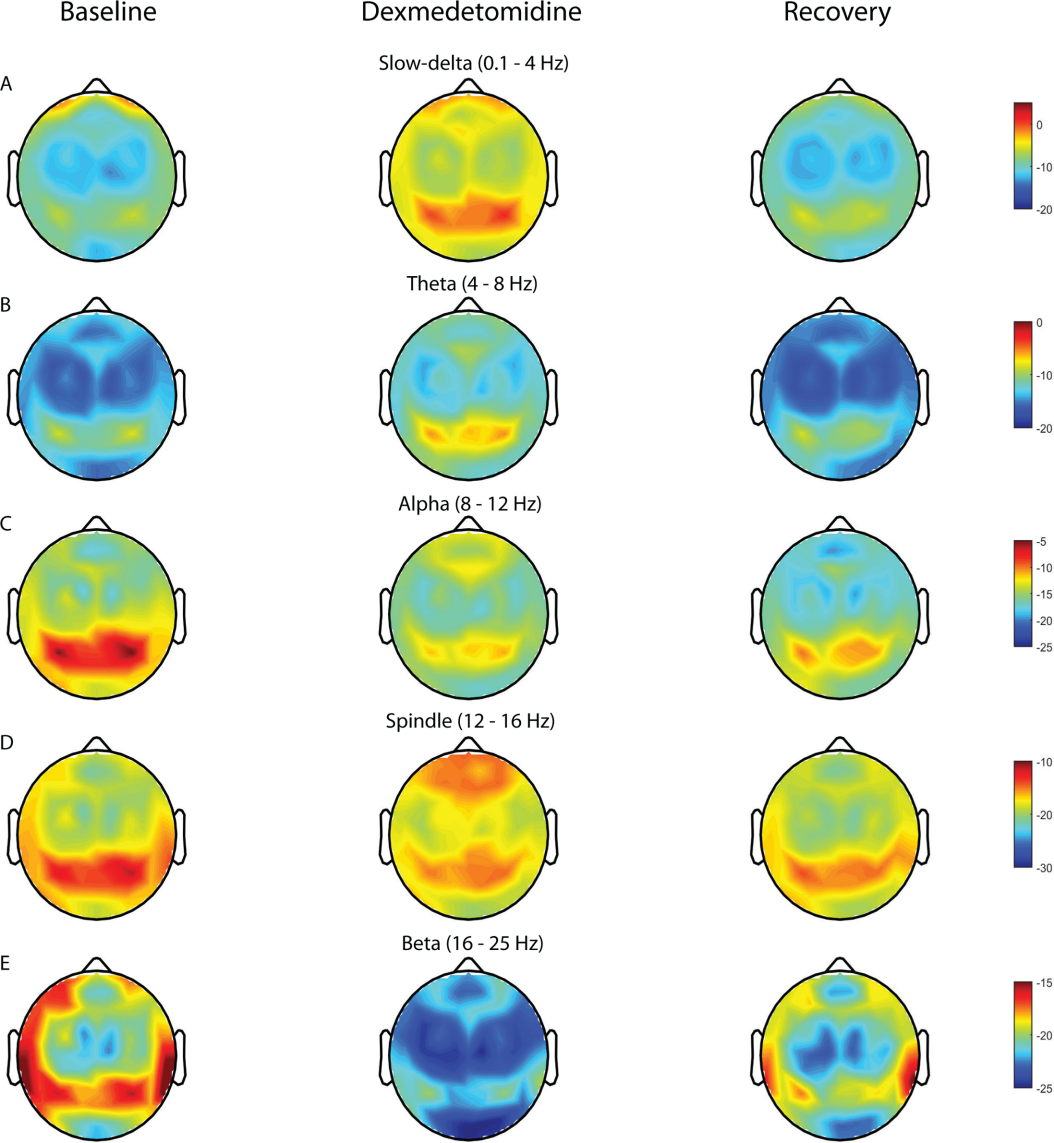
Topographic electroencephalogram maps of spectral power for distinct frequency bands. Topographic electroencephalogram maps detailing group-averaged power for each brain state we studied in frequency bands. (A) Slow-delta (0.1–4 Hz). (B) Theta (4–8 Hz). (C) Alpha (8–12 Hz). (D) Spindle (12–16 Hz). (E) Beta (16 – 25Hz). Dexmedetomidine is associated with increased slow-delta power with frontal/occipital dominance, increased occipital theta power, decreased occipital alpha power, increased fronto-central spindle power, and decreased beta power.

Group-median spectrograms were computed for 44 scalp electrode locations (Fig 1A, 1B and 1C). During the dexmedetomidine-induced brain state, we found increased slow-delta (0.1–4 Hz) oscillations in all electrodes, increased theta (4–8 Hz) oscillations in occipital electrodes, spindle oscillations (12–16 Hz) in frontal electrodes, and decreased beta (16–25 Hz) oscillations in all electrodes. To more clearly illustrate these findings, we computed topographic plots of power for slow-delta, theta, alpha, spindle, and beta frequency bands (Fig 2A, 2B, 2C, 2D and 2E). Group level median spectra at each electrode position with 95% confidence interval are presented in S1 Fig. As expected, we found the well-described occipital alpha oscillation during the baseline state because study volunteers were lying awake with their eyes closed. During the dexmedetomidine-induced brain state, instead of occipital alpha oscillations, we noticed occipital theta oscillations. Also, increased slow-delta oscillations appeared most predominant in frontal and occipital electrodes, increased spindle oscillations in frontal electrodes and decreased beta oscillations in all electrodes.

**Fig 3:**
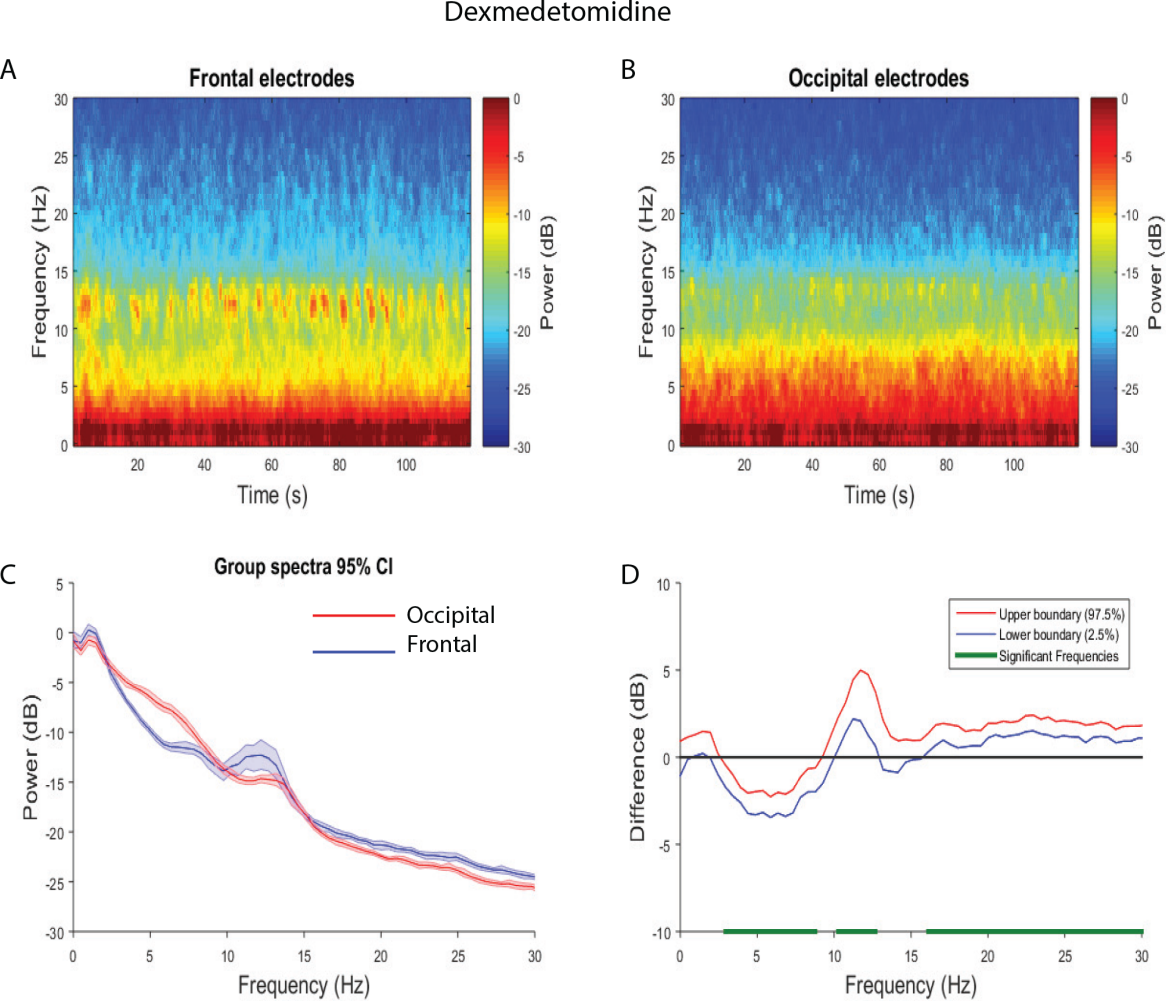
Spectral comparison of Frontal vs. Occipital electrodes during the dexmedetomidine-induced state. (A, B) Median frontal and occipital spectrograms during the dexmedetomidine-induced state (n = 8). (C) Overlay of median occipital spectrum (red), and median frontal spectrum (blue). Bootstrapped median spectra are presented and the shaded regions represent the 95% confidence interval for the uncertainty around each median spectrum. (D) The upper (red) and lower (blue) represent the bootstrapped 95% confidence interval bounds for the difference between spectra shown in panel C. We found that there were differences in power between frontal and occipital electrodes during the dexmedetomidine-induced state (frontal > occipital, 10.3–12.7 Hz, 16.1–30 Hz; occipital > frontal, 2.9–8.8 Hz).

We evaluated the frontal versus occipital dominance of the neural oscillations by assessing power spectra differences between frontal and occipital electrodes. During the baseline period, we found that slow-delta (0.5–2.9 Hz) and beta (23.4–30 Hz) oscillations were larger in the frontal region, while theta, alpha, and low beta (6.4–17.6 Hz) oscillations were larger in the occipital region (S2 Fig). Upon the administration of dexmedetomidine, when volunteers did not respond to the auditory task, we found that slow-delta and theta (2.9–8.8 Hz) frequency oscillations were larger in the occipital region. We also found that alpha, spindle, and beta oscillations were larger in the frontal region (Fig 3A, 3B, 3C and 3D; 10.3–12.7 Hz, 16.1–30 Hz). Thus, the dexmedetomidine-induced brain state was associated with a shift to occipital dominance of slow-delta oscillations, occipital theta oscillations, and frontal dominance of alpha/spindle oscillations. During the recovery period, we found larger slow-delta, theta, alpha (2.4–17.6 Hz) oscillations in the occipital region. We also found increased beta (20.5–30 Hz) power in the frontal region (S3 Fig). Thus, the recovery brain state was associated with occipital slow-delta oscillations, occipital theta oscillations, occipital alpha oscillations, and increased beta oscillations in the frontal region.

### Frontal and Occipital Electrode Power Spectral Comparisons across Brain States

**Fig 4:**
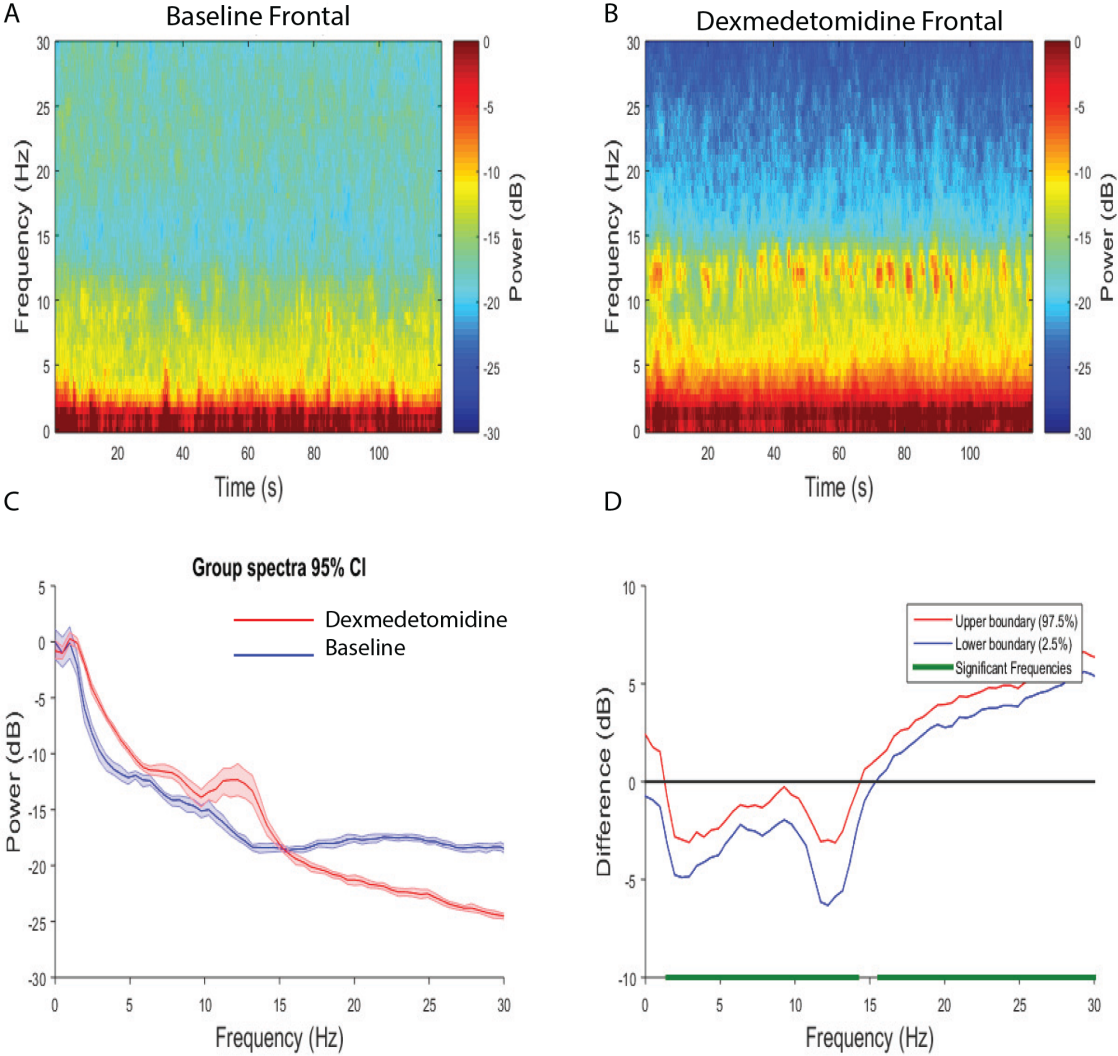
Spectral comparison of Baseline vs. Dexmedetomidine frontal electrodes. (A, B) Median frontal spectrograms (n = 8). (C) Overlay of median dexmedetomidine frontal spectrum (red), and median baseline frontal spectrum (blue). Bootstrapped median spectra are presented and the shaded regions represent the 95% confidence interval for the uncertainty around each median spectrum. (D) The upper (red) and lower (blue) represent the bootstrapped 95% confidence interval bounds for the difference between spectra shown in panel C. We found that there were differences in power between baseline and dexmedetomidine frontal electrodes (dexmedetomidine > baseline, 1.5–14.2 Hz; baseline > dexmedetomidine, 15.6–30 Hz).

**Fig 5:**
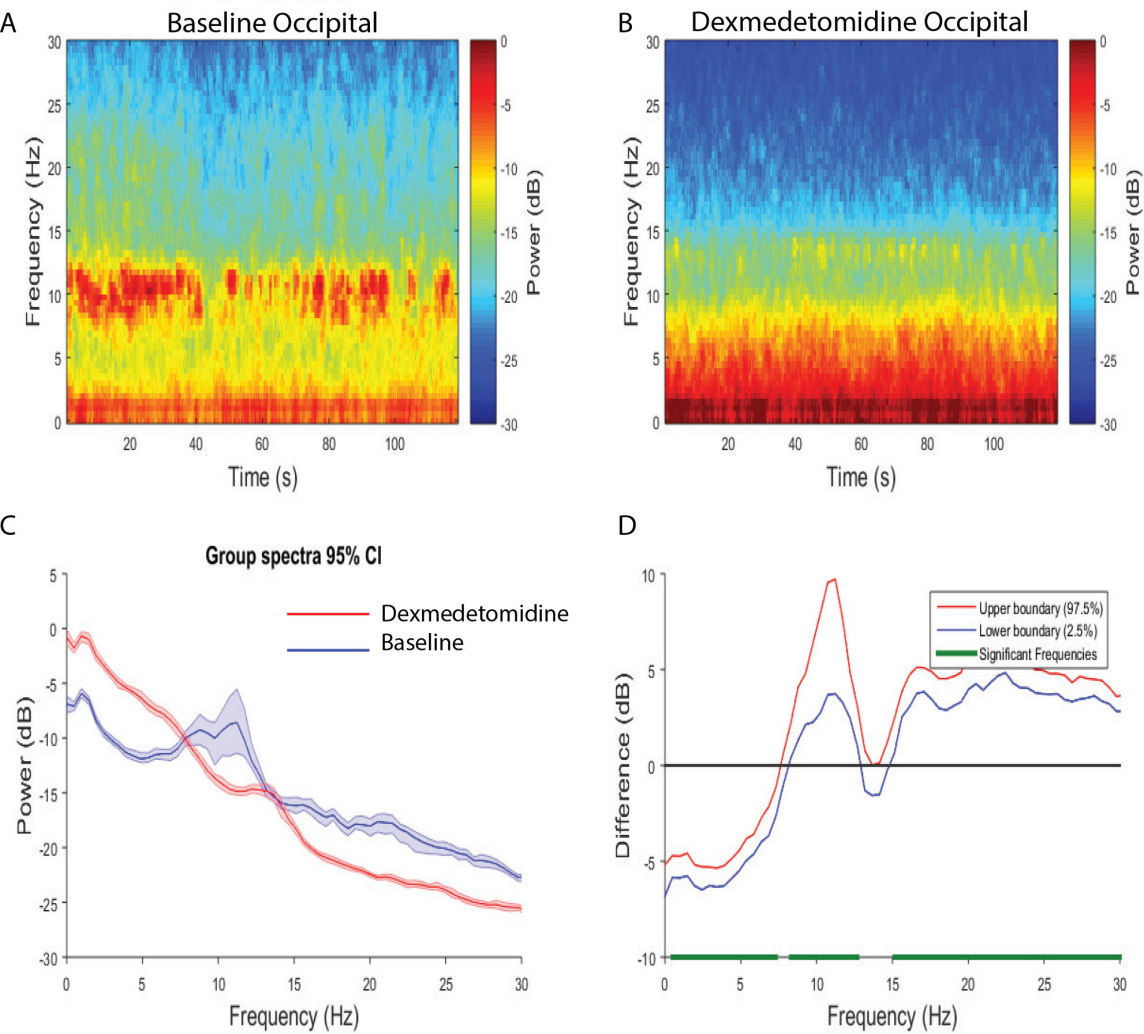
Spectral comparison of Baseline vs. Dexmedetomidine occipital electrodes. (A, B) Median occipital spectrograms (n = 8). (C) Overlay of median baseline occipital spectrum (red), and median dexmedetomidine occipital spectrum (blue). Bootstrapped median spectra are presented and the shaded regions represent the 95% confidence interval for the uncertainty around each median spectrum. (D) The upper (red) and lower (blue) represent the bootstrapped 95% confidence interval bounds for the difference between spectra shown in panel C. We found that there were differences in power between baseline and dexmedetomidine occipital electrodes (dexmedetomidine > baseline, 0.5–7.3 Hz; baseline > dexmedetomidine, 8.3–12.7 Hz, 15.6–30 Hz).

We evaluated power spectra differences between frontal versus frontal and occipital versus occipital electrodes across brain states. We found that the dexmedetomidine-induced brain state exhibited larger frontal power in slow-delta, theta, alpha and spindle (1.5–14.2 Hz) frequency bands, and decreased power in the beta (15.6–30 Hz) frequency band when compared to baseline (Fig 4). This finding was similar when we compared the dexmedetomidine state to recovery (S4 Fig; dexmedetomidine > recovery, 0.5–15.1 Hz; dexmedetomidine < recovery 17–30 Hz). We found that the dexmedetomidine-induced brain state exhibited larger occipital power in slow-delta, and theta (0.5–7.3 Hz) frequency bands, and decreased power in the alpha and beta (8.– 12.7 Hz, 15.6–30 Hz) frequency bands when compared to baseline (Fig 5). This finding was similar when we compared the dexmedetomidine state to recovery, however power changes within the alpha band did not reach statistical significance (S5 Fig; dexmedetomidine > recovery, 0.5–7.8 Hz; dexmedetomidine < recovery 15.1–30 Hz). Thus, although the dexmedetomidine-induced brain state is associated with increased slow-delta (frontal/occipital), theta (frontal/occipital), spindle (frontal), the increased occipital theta power was most unique to altered arousal.

### Global Coherence Analysis

**Fig 6:**
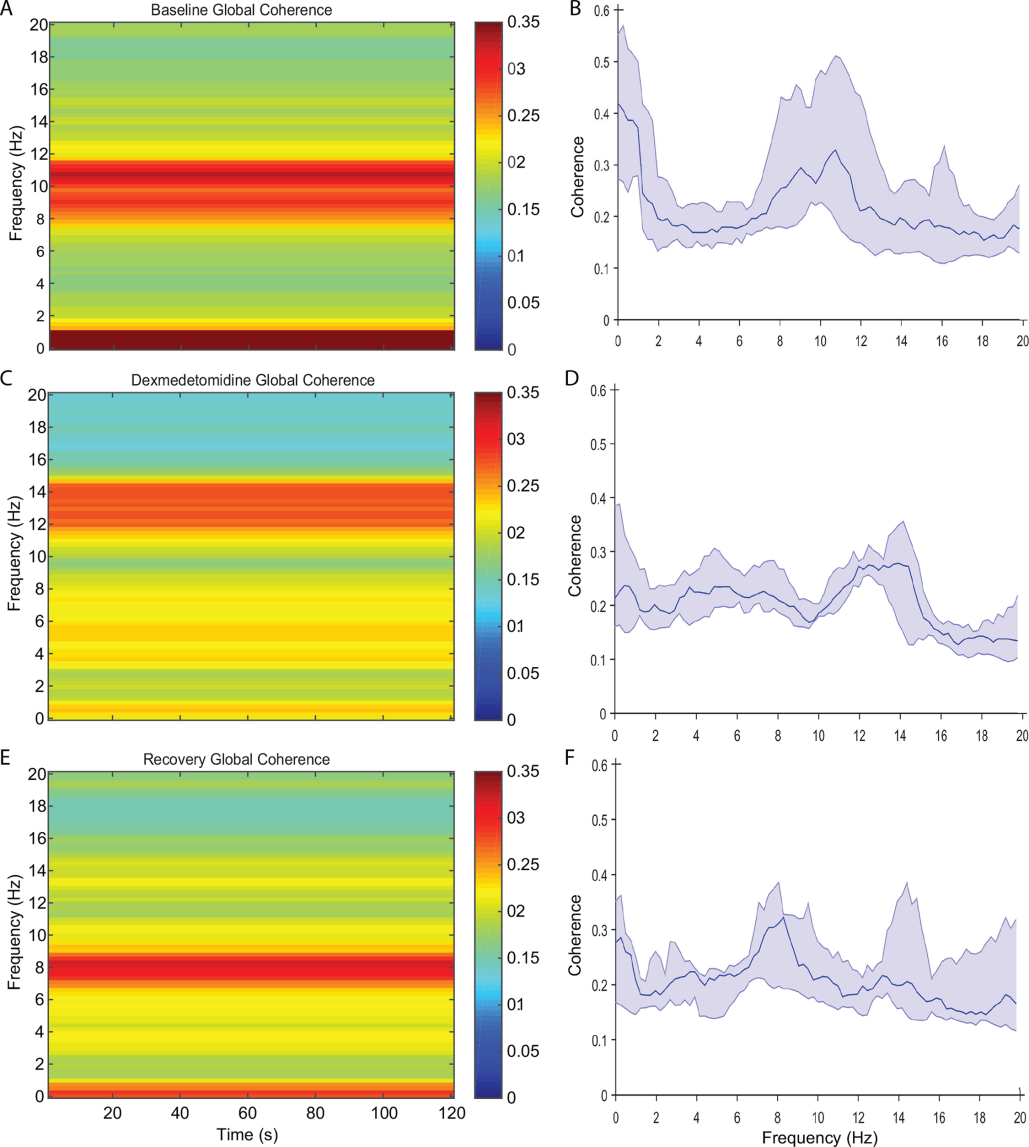
Global coherence analysis of baseline, dexmedetomidine-induced, and recovery brain states. (A,B) Globally coherent alpha oscillations centered at ~10 Hz can be observed during the baseline state. Also a globally coherence band at ~1 Hz is present. (C,D) Globally coherent spindle oscillations centered at ~14 Hz can be observed during the baseline state. The globally coherent dynamic at 1 Hz from A, B above is less coherent. Instead, there is increased global coherence in the theta frequency band. (E,F) Globally coherent alpha oscillations centered at ~8 Hz can be observed during the recovery state along with increased global coherence at ~ 1 Hz.

**Fig 7:**
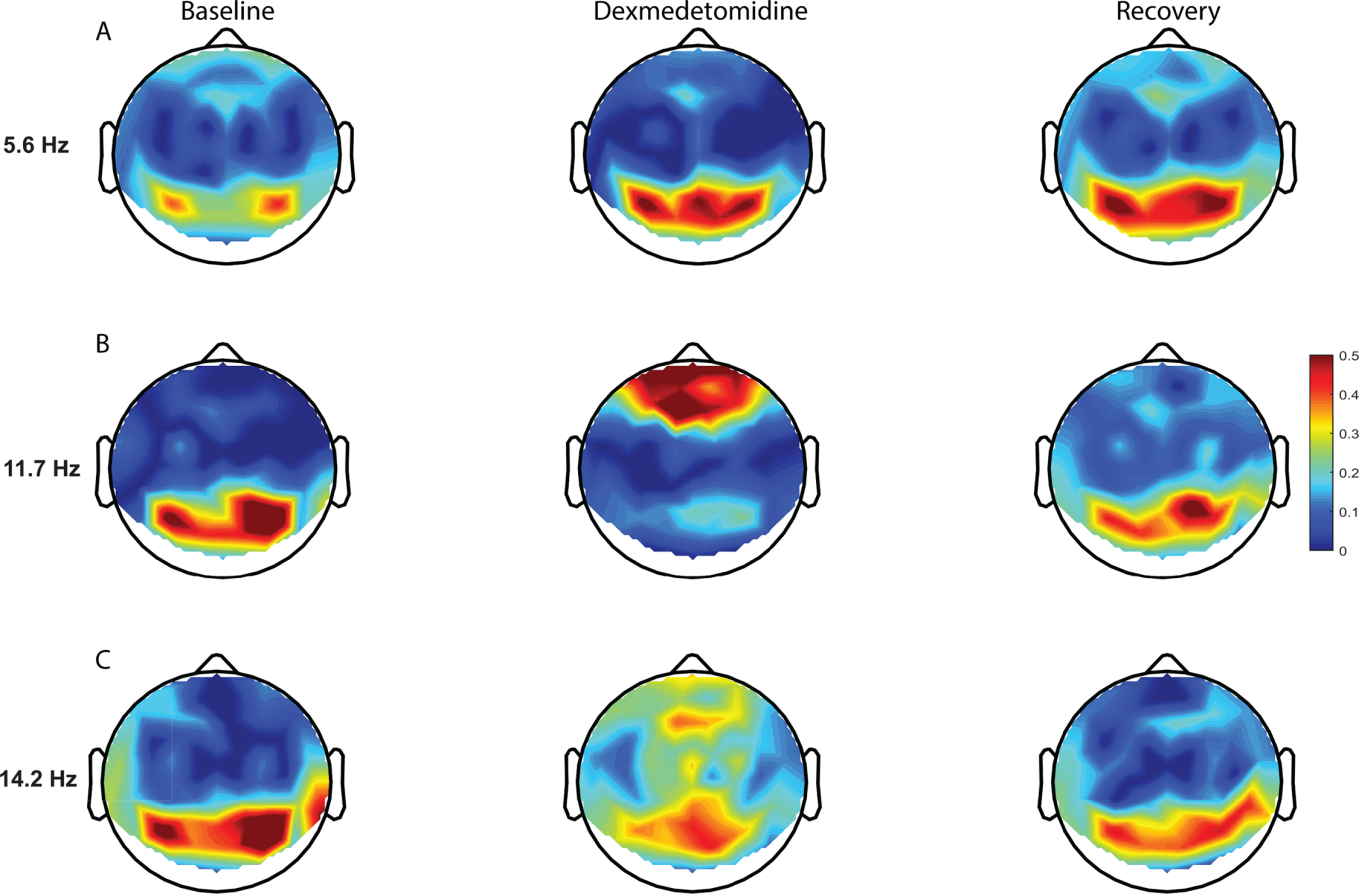
Topographic electroencephalogram maps detailing group-averaged global coherence for each electroencephalogram frequency of interest. (A) Dexmedetomidine is associated with increased occipital theta global coherence. (B) Dexmedetomidine is associated with a shift in the globally coherent occipital awake alpha to frontal regions. (C) Dexmedetomidine is associated with globally coherent fronto-central spindle oscillations.

Global coherence characterizes coordinated activity across multiple channels of the electroencephalogram as a function of frequency. It is the fraction of variance at a given frequency across all electroencephalogram channels explained by the first eigenvector of the cross-spectral matrix. The minimum value is attained when the readings from all electrode sites are random, whereas the maximum is attained when they are completely coherent. Thus, a large value of the global coherence suggests coordinated activity. We examined the group global coherence during the three states across time and found that the baseline and recovery states were associated with highly coordinated activity at approximately 10 Hz, whereas the dexmedetomidine induced brain state was associated with highly coordinated activity at approximately 5.7Hz and 14 Hz (Fig 6A, 6B, 6C, 6D, 6E and 6F). We performed modal projections (Fig 7) of the eigenvectors corresponding the largest eigenvalues for each state. During the dexmedetomidine state, we found coordinated theta activity in the occipital region (Fig 7A). The modal projections of the baseline state showed that the highly coordinated alpha oscillations are local to the occipital region (Fig 7B). The modal projection of the dexmedetomidine-induced brain state showed that the highly coordinated spindle oscillations are local to fronto-central regions (Fig 7C). Although theta oscillation power was decreased in the occipital region during the recovery period, the theta oscillations remained coherent (Fig 7A).

## Discussion

In this investigation we performed a systematic characterization of the spatiotemporal electroencephalogram dynamics associated with dexmedetomidine. We briefly summarize our findings as follows: 1) increased slow-delta oscillations across the entire scalp; 2) increased occipital theta oscillations; 3) increased fronto-central spindle oscillations; 4) decreased beta oscillations across the entire scalp, and; 5) globally coherent theta and spindle oscillations. These findings closely approximate the electroencephalogram dynamics described for human sleep onset (non-REM II) [14].

### The Occipital Theta Oscillation of Sleep and Dexmedetomidine

To our knowledge, the occipital theta oscillations we describe in this manuscript have never been attributed to any anesthetic-induced brain state, and remain pharmacologically unique to dexmedetomidine. This is not surprising because dexmedetomidine is specific to the adrenergic arousal circuit implicated in sleep onset, while other anesthetics affect brain circuits more globally. A synthesis of previous works suggests that occipital theta oscillations are only associated with physiological states having a high sleep pressure such as sleep deprivation [20], recovery sleep [21], and sleep onset [14, 22]. More specifically, intracortical recordings obtained in a patient with epilepsy suggest that theta is the main oscillatory activity of the occipital cortex during sleep onset [14]. Thus, the dexmedetomidine-induced brain state and the recovery period (with residual drug effects) likely reflect a brain state with high sleep pressure. This particular finding suggests that dexmedetomidine shares closer similarities with sleep than the current standard-of-care sedatives and anesthetic agents administered in clinical settings.

Hippocampal theta oscillations have been described during REM sleep in rodent models [8], and evidence suggests that these theta oscillations are associated with encoding of information and the modification of synaptic weights [23, 24]. However, the human equivalent oscillations as slower, occupying the delta frequency range [25]. Thus, the occipital theta oscillations we describe are likely distinct from hippocampal theta oscillations. At present, we cannot speculate on the functional relevance of sleep and dexmedetomidine-induced theta oscillations. However, this globally coherent oscillatory dynamic may be further studied to gain insights into both sleep and dexmedetomidine neural circuit dynamics.

### Benefits of Maintaining a Dexmedetomidine-Induced Altered Arousal State in Critically Ill Patients

Recently, two ICU polysomnography studies demonstrated that a continuous infusion of dexmedetomidine maintains patients in a brain state that was clinically scored as a non-REM II sleep state [26, 27]. Notably, sleep state switching into non-REM III or REM sleep was not observed [26, 27]. This is consistent with the putative mechanism of drug action, suggesting that an infusion of dexmedetomidine engages and sustains the mechanisms necessary for non-REM II sleep. Although associated with increased incidence of bradycardia and hypotension, results from clinical trials suggest that dexmedetomidine is associated with decreased time on mechanical ventilation [28–30], decreased ICU length of stay [28, 31], and lower rates of delirium [29, 30, 32]. We suggest that some of the benefits attributable to dexmedetomidine may result from its close approximation to a sleep state.

Although, the exact mechanisms of the dexmedetomidine-induced decreases in delirium are unknown, they may result from decreased levels of inflammatory mediators [33]. Conditions associated with delirium are characterized by activation of the inflammatory cascade with acute release of inflammatory mediators into the bloodstream [34–48]. Studies on healthy human volunteers exposed to bacterial endotoxin or lipopolysaccharide confirm that the inflammatory cascade is associated with deficits in cognitive function [49, 50]. A putative mechanism is the high interleukin-6 levels associated with delirium in laboratory animals [51–53] and humans [47, 54, 55]. Neuro-inflammation is exacerbated by sleep disturbances [56, 57], and pharmacologically maintained sleep states might be a modifiable risk factor for the development of delirium. Thus the close approximation of the dexmedetomidine-induced brain state to a physiological brain state may be associated with molecular and biochemical changes that confer some of the anti-inflammatory benefits of natural sleep.

Although the brain state induced by dexmedetomidine closely approximates a physiological brain state, maintaining patients for a prolonged period in the dexmedetomidine-induced brain state may not confer all the benefits of natural sleep. This is because maintaining a patient in a non-REM II state may not confer benefits that are specific to non-REM III and REM sleep stages. A recent laboratory investigation has confirmed previous observations in humans that dexmedetomidine eliminated REM sleep in rats, and that it may not fully compensate for sleep need [58]. We suggest a principled approach where the administration of dexmedetomidine in critically ill patients may involve different daytime and nighttime sedation drug regimens, and drug delivery systems with electroencephalogram feedback control to more precisely target brain-state goals of care. Such a drug delivery system may incorporate medications with REM enhancing properties such as rivastigmine and donepezil to achieve non-REM-REM cycling.

### Limitations

The electroencephalogram recordings analyzed in this paper were obtained from a high-density study. However, our analysis was limited to the sensor space. Therefore, we cannot make inferences on brain regions (i.e. cortical and subcortical) that may be integral for the oscillatory dynamics we describe. Also, spectral leakage is associated with spectral estimation methods. Thus, our results are limited by our spectral resolution. Further, even though the neural oscillatory “syntax” of dexmedetomidine is similar to non-REM stage II sleep, the molecular and biochemical characteristics of these brain states may differ. Future studies incorporating electroencephalogram source localization, intracortical recordings, and microdialysis techniques in human, non-human primates, and rodent models are necessary.

### Conclusion

The electroencephalogram dynamics induced by dexmedetomidine more closely approximates non-REM II sleep than previously appreciated. Therefore, incorporating dexmedetomidine into ICU sedation regimens when appropriate may confer some benefits of natural sleep compared to antipsychotic (i.e. haloperidol, seroquel) and sedative medications (i.e. midazolam, propofol). Furthermore, this quantitative analysis of dexmedetomidine-induced oscillations will inform mathematical modeling approaches that will further refine our mechanistic understanding of this brain state. This understanding may aid the development of next-generation anesthetic medications that approximate normal human neurophysiology with more limited side-effect profiles.

## Supporting Information

S1 Fig Group level median spectra at each electrode position with 95% confidence intervals. (A) Overlay of baseline and dexmedetomidine. (B) Overlay of baseline and recovery.
(EPS)

S2 Fig Spectral comparison of Frontal vs. Occipital electrodes during the baseline state. (A, B) Median frontal and occipital spectrograms during the baseline state (n = 8; within subject comparison). (C) Overlay of median occipital spectrum (red), and median frontal spectrum (blue). Bootstrapped median spectra are presented and the shaded regions represent the 95% confidence interval for the uncertainty around each median spectrum. (D) The upper (red) and lower (blue) represent the bootstrapped 95% confidence interval bounds for the difference between spectra shown in panel C. We found that there were differences in power between frontal and occipital electrodes during the baseline state (frontal > occipital, 0.4882–2.9297 Hz, 23.4375–30 Hz; occipital > frontal, 6.3477–17.5781 Hz).
(EPS)

S3 Fig Spectral comparison of Frontal vs. Occipital electrodes during recovery. (A, B) Median frontal and occipital spectrograms during the recovery state (n = 8; within subject comparison). (C) Overlay of median occipital spectrum (red), and median frontal spectrum (blue). Bootstrapped median spectra are presented and the shaded regions represent the 95% confidence interval for the uncertainty around each median spectrum. (D) The upper (red) and lower (blue) represent the bootstrapped 95% confidence interval bounds for the difference between spectra shown in panel C. We found that there were differences in power between frontal and occipital electrodes during the recovery state (frontal > occipital, 20.5–30 Hz; occipital > frontal, 2.4–17.6 Hz).
(EPS)

S4 Fig Spectral comparison of Dexmedetomidine vs. Recovery frontal electrodes. (A, B) Median frontal spectrograms (n = 8). (C) Overlay of median recovery frontal spectrum (red), and median dexmedetomidine frontal spectrum (blue). Bootstrapped median spectra are presented and the shaded regions represent the 95% confidence interval for the uncertainty around each median spectrum. (D) The upper (red) and lower (blue) represent the bootstrapped 95% confidence interval bounds for the difference between spectra shown in panel C. We found that there were differences in power between dexmedetomidine and recovery frontal electrodes (dexmedetomidine > recovery, 0.5–15.1 Hz; dexmedetomidine < recovery 17–30 Hz).
(EPS)

S5 Fig Spectral comparison of Dexmedetomidine vs. Recovery occipital electrodes. (A, B) Median frontal spectrograms (n **=** 8). (C) Overlay of median recovery occipital spectrum (red), and median dexmedetomidine occipital spectrum (blue). Bootstrapped median spectra are presented and the shaded regions represent the 95% confidence interval for the uncertainty around each median spectrum. (D) The upper (red) and lower (blue) represent the bootstrapped 95% confidence interval bounds for the difference between spectra shown in panel C. We found that there were differences in power between dexmedetomidine and recovery frontal electrodes (dexmedetomidine > recovery, 0.5–7.8 Hz; dexmedetomidine < recovery 15.1–30 Hz).
(EPS)
